# Empirical estimation of sequencing error rates using smoothing splines

**DOI:** 10.1186/s12859-016-1052-3

**Published:** 2016-04-22

**Authors:** Xuan Zhu, Jian Wang, Bo Peng, Sanjay Shete

**Affiliations:** Department of Biostatistics, The University of Texas MD Anderson Cancer Center, Houston, TX 77030 USA; Department of Bioinformatics & Computational Biology, The University of Texas MD Anderson Cancer Center, Houston, TX 77030 USA; Department of Epidemiology, The University of Texas MD Anderson Cancer Center, Houston, TX 77030 USA

**Keywords:** Empirical error rate, Next-generation sequencing, Smoothing spline, Frequency-based simulation, Short reads

## Abstract

**Background:**

Next-generation sequencing has been used by investigators to address a diverse range of biological problems through, for example, polymorphism and mutation discovery and microRNA profiling. However, compared to conventional sequencing, the error rates for next-generation sequencing are often higher, which impacts the downstream genomic analysis. Recently, Wang et al. (BMC Bioinformatics 13:185, 2012) proposed a shadow regression approach to estimate the error rates for next-generation sequencing data based on the assumption of a linear relationship between the number of reads sequenced and the number of reads containing errors (denoted as shadows). However, this linear read-shadow relationship may not be appropriate for all types of sequence data. Therefore, it is necessary to estimate the error rates in a more reliable way without assuming linearity. We proposed an empirical error rate estimation approach that employs cubic and robust smoothing splines to model the relationship between the number of reads sequenced and the number of shadows.

**Results:**

We performed simulation studies using a frequency-based approach to generate the read and shadow counts directly, which can mimic the real sequence counts data structure. Using simulation, we investigated the performance of the proposed approach and compared it to that of shadow linear regression. The proposed approach provided more accurate error rate estimations than the shadow linear regression approach for all the scenarios tested. We also applied the proposed approach to assess the error rates for the sequence data from the MicroArray Quality Control project, a mutation screening study, the Encyclopedia of DNA Elements project, and bacteriophage PhiX DNA samples.

**Conclusions:**

The proposed empirical error rate estimation approach does not assume a linear relationship between the error-free read and shadow counts and provides more accurate estimations of error rates for next-generation, short-read sequencing data.

**Electronic supplementary material:**

The online version of this article (doi:10.1186/s12859-016-1052-3) contains supplementary material, which is available to authorized users.

## Background

Next-generation sequencing usually refers to massively parallel high-throughput DNA sequencing technologies that can sequence millions of small DNA fragments at the same time [1, 2]. Next-generation sequencing has developed rapidly and is used in a diverse range of biological investigations, such as quantification of gene expression, polymorphism and mutation discovery, microRNA profiling, and genome-wide mapping of protein-DNA interaction [3–8]. Next-generation sequencing costs much less than conventional Sanger sequencing techniques because its high-throughput capacity enables a much higher degree of parallelism and much smaller reaction volumes [7, 9]. On the other hand, next-generation sequencing produces large numbers of short-read-length sequences [10, 11], which are difficult to correctly and fully assemble [12]. In addition, the error rates for next-generation sequencing are often higher than those for conventional Sanger sequencing, which negatively impacts downstream genomic analyses that use next-generation sequencing data [7, 9, 13, 14]. Therefore, before genomic analyses can be performed, quality assessment of the next-generation sequencing reads is essential [15]. These assessments include measuring intrinsic quality metrics (FastQC) [16], sequence coverage, sequence error rates, and paired-end, fragment-size distributions [15, 17].

Different approaches to estimating sequence error rates have been proposed, with or without the use of a reference genome. Bullard et al. [18] aligned the sequencing reads to a reference genome to obtain the numbers of uniquely mapped reads with 0, 1, or 2 mismatches, and the per-read sequencing error rate was calculated as the proportion of reads with at least one error. This mismatch counting approach assumed that reads with 0 mismatch had no errors and reads with 1 or 2 mismatches were errors but not polyphorphisms [7]. Also, this approach depends on a well-established reference, which might not be available when sequencing a new genome [17]. The error correction tools based on *k*-mer [15, 19–25] can also provide estimations for sequencing error rates. These approaches are reference free and less sensitive to errors that occur due to polymorphisms [26]. However, since the *k*-mer-based approaches usually involve computing the frequencies of all distinct sub-strings of length *k* appearing in the sequence, these approaches are computationally demanding and require a large amount of computer memory, making it difficult for them to process reads from large genomes [27–29].

Recently, Wang et al. [7] developed a novel approach called shadow regression to estimate short sequencing read error rates without a reference genome. These authors observed that the number of shadows due to sequencing errors increases linearly with the number of reads sequenced. The per-read error rate was defined as the proportion of reads containing sequencing errors among all the reads in a sample. A linear regression model was employed to assess the error rates (details will be reviewed in the Method section). However, in practice, the linear assumption for the relationship between the number of reads sequenced and the number of reads that contain errors might not be appropriate for all types of sequence data (see the sample data from [7], Additional file 1). In these situations, the shadow regression based on a linear assumption might lead to a biased estimation of the sequencing error rates.

Therefore, in this study, we proposed an empirical approach to estimate the sequencing error rates that uses cubic smoothing spline and robust smoothing spline methods to model the relationship between the number of reads sequenced and the number of reads that contain errors. We took the per-read error rate used by Wang et al. [7] and redefined it as a function of the read counts. We also defined a sample per-read error rate as the median of the per-read error rates obtained using different numbers of read counts and corresponding fitted counts of reads containing errors. To better investigate the performance of the proposed approach, we developed a frequency-based simulation approach based on the sequencing reads from real data sets that mimics the non-linear relationship between the numbers of reads sequenced and the numbers of reads that contain errors more realistically than the Wang et al.’s simulation approch. We performed the simulation studies using sequence sample data from the MicroArray Quality Control (MAQC) project [30], a mutation screening study [31], the Encyclopedia of DNA Elements (ENCODE) project [32], and bacteriophage PhiX 174RF1 DNA samples [7] and compared the results with those obtained by using shadow linear regression. The proposed empirical approach provided more accurate error rate estimations than shadow linear regression for all the scenarios in which the assumption of linearity was not valid and provided similar error rates when the assumption of linearity was valid. We also applied the empirical error rate estimation approach to assess the sequencing error rates for real sample data from MAQC, mutation screening, ENCODE, and PhiX DNA samples.

## Methods

### Shadow regression overview

Wang et al. [7] made the important observation that the number of shadows due to sequencing errors increases linearly with the number of reads sequenced, whereas the number of true shadows is independent of the number of reads sequenced. In the current study, we employed and modified the definitions and notations from the Wang et al. [7] study.

Specifically, given a sequence *t* in a sample, the total number of reads can be given as *r*_*t*_ = *n*_*t*_ + *e*_*t*_, where, *r*_*t*_ is the total number of reads with sequence *t*, *n*_*t*_ is the number of reads that are error free with sequence *t*, and *e*_*t*_ is the number of reads that contain sequencing errors with sequence *t*. In practice, one would not know the true error-free sequence reads. Wang et al. first converted a sequence file (i.e., FASTQ format with equal length for all reads) from a sample into a read counts file. The number of reads for each sequence was counted over the sample, and then the authors ranked all the sequences according to the read counts (see an example of read counts in Additional file 2). The top 1000 reads with the highest frequencies in a sample were selected as the error-free sequences and used to calculate *n*_*t*_ and *e*_*t*_ in a different sequence *t*. The shadows of a given sequence *t* were defined as the reads differing from the error-free reads by up to two bases, which was deemed by Wang et al. [7] to be sufficiently similar to the given error-free sequence *t* to estimate substitution errors. Finally, the top 1000 reads with the highest frequencies in a sample were excluded from the assessment of the shadow counts.

The per-read error rate was defined as the proportion of reads containing sequencing errors over all the reads in a given sample [7], or $ER = \frac{{\displaystyle {\sum}_t}{e}_t}{{\displaystyle {\sum}_t}{r}_t}=\frac{\Delta {e}_t}{\Delta {r}_t}.$ Based on the observation that the number of shadows due to the sequencing errors increases linearly with the number of reads, Wang et al. proposed a linear model as *s*_*t*_ = *α* + *βn*_*t*_ + *ϵ*, where *s*_*t*_ is the number of shadows of sequence *t* and *ϵ* is the independent error that follows approximately Gaussian distribution. Robust linear regression was used to estimate the coefficients *α* and *β* because shadows can come from legitimate error-free reads. In this situation, the per-read error rate can be estimated by the slope of the linear model as $ER=\frac{\Delta {e}_t}{\Delta {r}_t}=\frac{\Delta {e}_t}{\Delta {n}_t+\Delta {e}_t}=\frac{\beta }{1+\beta },$ which was denoted as the shadow regression error rate (SRER) in the current study.

### Empirical per-read error rate with smoothing splines

The error rates assessed by shadow linear regression were based on the assumption of a linear relationship between the error-free read counts and shadow counts. However, the real data examples in Wang et al.’s paper (e.g., Additional file 1) show that for many types of sequencing data the relationship between error-free read counts and shadow counts does not follow a linear trend. In these situations, shadow regression based on a linear assumption might lead to a biased estimation of the sequence error rate. Therefore, in this study, we employed the cubic smoothing spline and robust smoothing spline methods to model the relationship between the error-free read counts and the shadow counts; the piecewise curve resulting from these spline methods is capable of capturing relationships of widely varying form and tends to avoid erratic behavior near the extremes of the data [33]. Based on the empirical read-shadow relationship, we proposed the error rate estimation as a function of error-free read counts, which we hypothesized would be more useful in practice than the shadow regression approach for estimating error rates.

We first used the cubic smoothing spline method [34, 35] to model the shadow counts (*s*_*t*_) as a function of the error-free read counts (*n*_*t*_). The cubic smoothing spline method fits a smooth curve to a set of observations using a cubic function [35, 36]. Specifically, given a set of observations of error-free read counts and shadow counts, (*n*_*1*_, *s*_*1*_), (*n*_*2*_, *s*_*2*_), …, (*n*_*m*_, *s*_*m*_), *n*_*1*_ *< n*_*2*_ *< … < n*_*m*_, we modeled the relationship between *n*_*i*_ and *s*_*i*_ as a function ${s}_i=\mathcal{U}\left({n}_i\right),$ with two continuous derivatives, where *n*_*i*_ is the number of error-free read counts with sequence *i*, *s*_*i*_ is the number of shadow counts with sequence *i*, *i* = 1, …, *m*, and *m* is the total number of sequences. Among all such functions with two continuous derivatives, the purpose of the smoothing spline is to find the estimated function minimizing the penalized residual sum of squares ${\varSigma}_1^m{\left({s}_i-\widehat{U}\left({n}_i\right)\right)}^2+\lambda {\displaystyle {\int}_{n_1}^{n_m}\widehat{U}}\;\mathit{\hbox{'}}\mathit{\hbox{'}}{(n)}^2dn,$ where *λ* is a smoothing parameter [35]. We used the cubic smoothing spline implemented in the R function “smooth.spline” in the “stats” package. To improve the robustness of the spline, we used a robust smoothing spline approach to model the relationship between shadow counts (*s*_*t*_) and error-free read counts (*n*_*t*_). This approach uses an iterative re-weighted smoothing spline algorithm with the inverse of the absolute value of the residuals as the weights. We used the robust smoothing spline implemented in the R function “robustSmoothspline” in the package “aroma.light” [37, 38].

Based on the fitted model of shadow counts as a spline function of error-free read counts, we used the definition of the per-read error rate used by Wang et al. [7]. Let *n*_*1*_, *n*_*2*_, …, *n*_*m*_ be the read counts of *m* sequences, where *n*_*1*_ < *n*_*2*_, …, *n*_*m-1*_ < *n*_*m*_, and let *Ŝ*_1_, *Ŝ*_2_, …, *Ŝ*_m_ be the corresponding fitted values of shadow counts using the smoothing spline approaches described above. Given a sequence *t* with read count *n*_*t*_ and fitted shadow count *Ŝ*_t_, we defined the per-read error rate as

$$ ER\left({n}_t\right)=\frac{{\displaystyle {\sum}_{j=1}^t}{\widehat{S}}_j}{{\displaystyle {\sum}_{j=1}^t}{\widehat{S}}_j+{\displaystyle {\sum}_{j=1}^t}{n}_j}. $$

Estimation of error rates using this definition also assumes the increasing number of shadows with the increasing number of read counts in the presence of sequencing errors, but this definition allows error rates to vary by read counts, whereas the Wang et al. [7] approach assumes the error rate to be a constant irrespective of the number of reads. For the purpose of comparison with the Wang et al. [7] approach, we also defined a sample per-read empirical error rate. Given a sample with *m* sequences, we randomly sampled 1000 numbers of read counts from *n*_*1*_ to *n*_*m*_ and calculated the per-read error rates for each of these read counts using the fitted spline. The median of these per-read error rates was reported as a sample per-read empirical error rate (henceforth denoted as EER).

### Next-generation sequencing data

We used next-generation sequencing data from four projects--the MicroArray Quality Control (MAQC) project (mRNA-seq) [30], a mutation screening study (re-sequencing) [31], the Encyclopedia of DNA Elements (ENCODE) project (mRNA-seq) [32], and bacteriophage PhiX 174RF1 (PhiX) DNA samples [7]--to perform the simulations and demonstrate the accuracy of the proposed empirical error rate estimation approach. PhiX DNA data in FASTQ format were generated by the Center for Cancer Computational Biology at Dana-Farber Cancer Institute and provided by Wang et al. [7]. The other three data sets are publicly available from the National Center for Biotechnology Information Sequence Read Archive [39] in FASTQ format with equal read lengths in each sample. All the data were converted to read counts using the shadow regression program provided by Wang et al. [7].

### MAQC brain experiment 2 data

The MAQC project was initiated to address concerns about the reliability of microarray technology [30]. This project provided gene expression levels measured from two RNA samples, including a Universal Human Reference RNA from Stratagene and a Human Brain Reference RNA from Ambion, in four titration pools on seven microarray platforms with three expression methodologies. In this study, we used the sequence data from the MAQC brain experiment 2 (Sequence Read Archive [SRA]010153), including 14 samples on two flowcells (SRX016366 and SRX016368) run on the Illumina 1G Genome Analyzer with each sample containing ~12 million reads.

### Mutation screening resequencing data

The mutation screening study provided re-sequencing data (SRA010105) from 24 patients with X-linked mental retardation (XLMR) for mutations in 86 previously identified XLMR genes, using a method that combined a novel droplet-based multiplex PCR method and next-generation sequencing [31]. An Illumina/Solexa Genome Analzer II platform was used to perform the sequencing, and each sample contained ~12 million reads.

### ENCODE transcriptome data

The ENCODE project used high-throughput approaches to provide a biologically more informative representation of the human genome [32]. The ENCODE pilot phase included more than 200 experimental and computational data sets from 35 groups [32]. In this study, we used the ENCODE human mRNA sequence data (SRA001150), including 5 samples of human cell line K562 (SRX000570) run on the Illumina 1G Genome Analyzer; each sample contained ~12 million reads.

### PhiX DNA data

The bacteriophage PhiX 174 is an icosahedral virus. It contains a closed circular single-stranded DNA molecule with 5386 nucleotide bases [40]. PhiX 174 was the first DNA-based genome for which the complete nucleotide sequence was successfully determined [40–42]. In this study, we used two PhiX DNA samples provided by Wang et al., which were generated from the Center for Cancer Computational Biology at Dana-Farber [7]. The PhiX DNA samples were sequenced using Illumina. One sample contained ~14.6 million reads, and the other contained ~25.7 million reads.

### Simulation approaches

We performed simulation studies to investigate the performance of the proposed EER and compare it with the SRER. We used two approaches to perform the simulation: (1) Wang et al.’s simulation approach and (2) a new frequency-based simulation approach described below.

### Wang et al. simulation approach

In the Wang et al. study [7], the simulation was conducted based on a sample from the MAQC brain experiment 2 (SRR037440) using calibration. The authors considered reads uniquely mapped to the reference genome with no mismatches as error-free reads and then added substitution errors based on pre-specified base-specific error rates, which were estimated from the sample SRR037440 by counting the number of mismatches to the reference genome at each base. To mimic the Wang et al. simulation procedure, we used the same sample (SRR037440) to retrieve the error-free reads and estimate the pre-specified base-specific error rates. In particular, we retrieved ~4.4 million perfectly matched reads from the SRR037440 sample, which originally had ~12 million reads. We used several approaches to estimate the pre-specified base-specific error rates. In our first approach, we aligned the reads to the reference genome, marked the mismatch locations, and then calculated base-specific error rates as a percentage of the mismatch nucleotides at each location (Additional file 3(C)). In our second approach, we located all unprocessed reads for each sequence, recorded locations and numbers of mismatch nucleotides, and calculated the base-specific error rates from the total number of mismatch nucleotides at each location (Additional file 3(D)). However, we found that neither of these approaches could obtain the same base-specific error rates shown in the Wang et al. study. Thus, we included the pre-specified base-specific error rates used in their study, which we extracted approximately from the figures in the article [7] (Additional file 3(E)). In addition, we used the base-specific error rates for sample SRR037440 obtained from the shadow regression software developed by Wang et al. [7] as another set of pre-specified base-specific error rates (Additional file 3(F)).

The Wang et al. simulation approach assumed an underlying linear relationship between the error-free read counts and shadow counts, no matter which base-specific error rate was used. Therefore, the data simulated by using the Wang et al. simulation approach would not reflect the non-linear relationships between error-free read counts and shadow counts (Additional file 3(A)). For example, in some samples, when the number of error-free read counts is low, the shadow counts may decrease as the error-free read counts increase. Therefore, we proposed a frequency-based simulation approach to generate the error-free read counts and shadow counts directly, which can mimic the real sequence counts of the data structure.

### Frequency-based simulation approach

Given the counts of all sequence reads in a real sample, we followed the same procedure described in Wang et al. to obtain the error-free read counts (*n*_*i*_) and shadow counts (*s*_*i*_) for *N* sequence reads, *i* = 1, 2, … *N* and *n*_*1*_ < *n*_*2*_, …, *n*_*N-1*_ < *n*_*N*_. We selected the top *N* reads with the highest frequencies to be the error-free reads and obtained the corresponding read counts (*n*_*i*_); we then mapped all the rest of the sequences in the sample to the error-free reads to identify the corresponding shadow counts (*s*_*i*_). We used *N* = 1000, as suggested in Wang et al. Based on the read counts and shadow counts in the real sample, we first constructed a frequency table for the error-free read counts (*n*_*i*_) using pre-specified bin widths. Because in most of the samples we investigated, the error-free read counts became very sparse when they were large, we used unequal bin widths to avoid having almost empty bins. Within each bin of the error-free read counts, we then constructed a frequency table for the shadow counts (*s*_*i*_) using pre-specified equal bin widths. Given *M* bins for error-free read counts and *L* bins for shadow counts within each of the error-free read count bins, the frequencies can be written as *p*_*j*_, *j* = 1, 2, …, *M*, for error-free read counts and *q*_*kj*_, *k* = 1, 2, …., *L* and *j* = 1, 2, …, *M*, for shadow counts. To sample a pair of observations (*n*_*new*_, *s*_*new*_), we first sampled two independent random numbers, *U* ~ uniform(0, 1) and *V* ~ uniform(0,1). If *U* ∈ [∑_*i* = 1_^*j*^*p*_*i*_, ∑_*i* = 1_^*j* + 1^*p*_*i*_), we sampled *n*_*new*_ ~ uniform(*n*_*j*_, *n*_*j+1*_), where *n*_*j*_ and *n*_*j+1*_ are the endpoints for the corresponding bin *j* of read counts. Further, within the read counts bin *j*, if *V* ∈ [∑_*i* = 1_^*k*^*q*_*ij*_, ∑_*i* = 1_^*k* + 1^*q*_*ij*_), we sampled *s*_*new*_ ~ uniform(*s*_*k*_, *s*_*k+1*_), where *s*_*k*_ and *s*_*k+1*_ are the endpoints for the corresponding bin *k* of shadow counts. With this approach, we can generate more pairs of error-free read and shadow counts (i.e., > 1000).

A comparison between the Wang et al. simulation approach and our proposed frequency-based simulation approach, using the SRR037440 sample, showed that unlike the frequency-based approach, the Wang et al. simulation approach does not mimic the observed non-linear data relationship between error-free read and shadow counts (Additional files 3 and 4). Therefore, we applied only the proposed frequency-based simulation approach in the simulation studies. We generated data based on the information from samples from the four data sets described above. For each sample, the median EER was calculated based on 1000 replicates and compared with the median SRER. For all the simulations, we considered the top 1000 reads of the sample of interest with the highest frequencies as the error-free reads and then determined the shadow counts accordingly, which were then used to generate the frequency tables for the simulations. Based on the frequency tables, we generated 1000 pairs of error-free reads and shadow counts for each replicate. For the simulation data sets, we also defined an expected per-read error rate as ∑_*i*_*s*_*i*_/(∑_*i*_*s*_*i*_ + ∑_*i*_*n*_*i*_), where *s*_*i*_ and *n*_*i*_ are the shadow count and error-free read count, respectively, for sequence *i*, *i* = 1, …, *M*. Note that *M* = 1000 in the simulation studies, as we generated 1000 pairs of error-free read and shadow counts.

## Results

### Simulation results

As shown in Additional files 3 and 4, using the frequency-based simulation approach can better capture the relationship between error-free read and shadow counts, therefore, we used this simulation approach to perform further simulations based on next-generation sequencing data from the MAQC, mutation screening, ENCODE, and PhiX DNA sample data sets. We compared the performance of our proposed EER approach using the cubic or robust smoothing spline method (EER_CS or EER_RS, respectively) with that of the SRER approach.

### Simulation results for MAQC data

Table 1 shows the median error rates (based on 1000 replicates) obtained using the shadow linear regression approach and the proposed smoothing spline approaches, based on the next-generation sequencing data from the MAQC study. We have also reported the expected error rates (calculated as described above) and the estimation biases, which were calculated as the absolute differences between the estimated error rates and the expected error rates. For all 14 samples of MAQC data, both smoothing spline approaches provided more accurate estimations of the error rates with less bias than the shadow linear regression approach. Both smoothing spline approaches performed very similarly. For example, for sample SRR037452, the median expected error rate in the simulation was 0.3305. Using SRER, the median estimated error rate was 0.2578, with a bias of 0.0727 compared to the expected error rate. In contrast, EER_CS and EER_RS had median estimated error rates of 0.3104 and 0.3096, respectively, with biases of 0.0201 and 0.0209, respectively. From these results, we can observe that the SRER approach underestimated the error rates, while the smoothing spline approaches provided more accurate estimated error rates.

**Table 1:** Median error rates in MAQC data using shadow linear regression and smoothing spline approaches^a^

Samples	Expected ER	SRER	SRER Bias	EER_CS	EER_CS Bias	EER_RS	EER_RS Bias
SRR037452	0.3305	0.2578	0.0727	0.3104	0.0201	0.3096	0.0209
SRR037453	0.1917	0.1584	0.0333	0.1824	0.0093	0.1818	0.0099
SRR037454	0.2354	0.1515	0.0839	0.2060	0.0294	0.2059	0.0295
SRR037455	0.1759	0.1448	0.0311	0.1675	0.0084	0.1668	0.0091
SRR037456	0.2312	0.1622	0.0690	0.2037	0.0275	0.2035	0.0277
SRR037457	0.1841	0.1480	0.0361	0.1777	0.0064	0.1771	0.0070
SRR037458	0.2653	0.2321	0.0332	0.2582	0.0071	0.2575	0.0078
SRR037459	0.2371	0.1943	0.0428	0.2202	0.0169	0.2203	0.0168
SRR037460	0.2530	0.2018	0.0512	0.2503	0.0027	0.2490	0.0040
SRR037461	0.2180	0.1704	0.0476	0.2105	0.0075	0.2104	0.0076
SRR037462	0.2443	0.1734	0.0709	0.2322	0.0121	0.2308	0.0135
SRR037463	0.2154	0.1654	0.0500	0.2023	0.0131	0.2045	0.0109
SRR037464	0.2624	0.1666	0.0958	0.2392	0.0232	0.2403	0.0221
SRR037465	0.2145	0.1742	0.0403	0.2038	0.0107	0.2037	0.0108

^a^Based on 1000 replicates. The frequency-based simulation approach was applied. For each replicate, we considered the top 1000 reads with the highest frequencies as the error-free reads and generated 1000 pairs of error-free read counts and shadow counts

*Expected ER* expected error rate in simulation studies

*SRER* error rate estimated using shadow regression

*SRER Bias* the absolute value of the difference between SRER and Expected ER

*EER_CS* empirical error rate estimated using cubic smoothing spline

*EER_CS Bias* the absolute value of the difference between EER_CS and Expected ER

*EER_RS* empirical error rate estimated using robust smoothing spline

*EER_RS Bias* the absolute value of the difference between EER_RS and Expected ER

### Simulation results for mutation screening resequencing data

Table 2 reports the median error rates obtained using the SRER approach and the two EER approaches, based on next-generation sequencing data from a mutation screening study. Similar to the results from the MAQC samples, all 24 mutation screening resequencing samples yielded error rates for the smoothing spline approaches that were more accurate than or comparable to the estimations of error rates for SRER. For example, in sample SRR032566, the median of the expected error rate in the simulation was 0.0734. Using SRER, the median of the estimated error rate was 0.0412, with a bias of 0.0323 compared with the expected error rate. Using the smoothing spline approaches, the median of the estimated error rate was 0.0705 for both approaches, with a very small bias of 0.0029.

**Table 2:** Median error rates in mutation screening data using shadow linear regression and smoothing spline approaches^a^

Samples	Expected ER	SRER	SRER Bias	EER_CS	EER_CS Bias	EER_RS	EER_RS Bias
SRR032565	0.1991	0.0493	0.1498	0.1080	0.0911	0.1082	0.0910
SRR032566	0.0734	0.0412	0.0323	0.0705	0.0029	0.0705	0.0029
SRR032567	0.2003	0.0542	0.1461	0.1111	0.0892	0.1111	0.0893
SRR032568	0.2040	0.0437	0.1603	0.1057	0.0984	0.1072	0.0968
SRR032569	0.1598	0.0509	0.1089	0.1018	0.0580	0.1014	0.0585
SRR032570	0.0985	0.0641	0.0345	0.0959	0.0026	0.0954	0.0032
SRR032571	0.1236	0.0575	0.0661	0.1406	0.0170	0.1406	0.0170
SRR032572	0.1495	0.0530	0.0965	0.1181	0.0314	0.1173	0.0323
SRR032573	0.1779	0.0912	0.0867	0.1518	0.0261	0.1506	0.0273
SRR032574	0.0986	0.0384	0.0602	0.0626	0.0361	0.0618	0.0368
SRR032575	0.1169	0.0839	0.0330	0.1228	0.0059	0.1227	0.0058
SRR032576	0.1554	0.0945	0.0609	0.1887	0.0333	0.1880	0.0326
SRR032577	0.1052	0.0694	0.0359	0.1054	0.0002	0.1055	0.0002
SRR032578	0.1143	0.0448	0.0695	0.1077	0.0067	0.1076	0.0067
SRR032580	0.0870	0.0619	0.0251	0.1169	0.0298	0.1163	0.0292
SRR032581	0.0770	0.0347	0.0424	0.0752	0.0018	0.0751	0.0019
SRR032582	0.0400	0.0041	0.0359	0.0309	0.0091	0.0310	0.0090
SRR032583	0.1224	0.0707	0.0517	0.1279	0.0055	0.1280	0.0056
SRR032584	0.1290	0.0540	0.0750	0.1052	0.0238	0.1032	0.0259
SRR032586	0.0445	0.0102	0.0343	0.0287	0.0158	0.0287	0.0159
SRR032587	0.1486	0.0786	0.0700	0.1562	0.0076	0.1552	0.0066
SRR032588	0.1240	0.0470	0.0770	0.1024	0.0216	0.1021	0.0220
SRR033543	0.1151	0.0587	0.0564	0.0828	0.0323	0.0832	0.0318
SRR033544	0.1267	0.0524	0.0743	0.1086	0.0181	0.1085	0.0182

^a^Based on 1000 replicates. The frequency-based simulation approach was applied. For each replicate, we considered the top 1000 reads with the highest frequencies as the error-free reads and generated 1000 pairs of error-free read counts and shadow counts

*Expected ER* expected error rate in simulation studies

*SRER* error rate estimated using shadow regression

*SRER Bias* the absolute value of the difference between SRER and Expected ER

*EER_CS* empirical error rate estimated using cubic smoothing spline

*EER_CS Bias* the absolute value of the difference between EER_CS and Expected ER

*EER_RS* empirical error rate estimated using robust smoothing spline

*EER_RS Bias* the absolute value of the difference between EER_RS and Expected ER

### Simulation results for ENCODE transcriptome data

Table 3 reports the median error rates obtained using different approaches based on next-generation sequencing data from the ENCODE study. The five samples from the ENCODE study had higher expected error rates than the samples in the MAQC and mutation screening studies, which might have been due to relatively large shadow counts that corresponded with smaller error-free read counts Additional file 1(C). In this situation, the smoothing spline approaches still performed better than shadow linear regression. For example, in sample SRR002056, the expected error rate was 0.3646, and the estimated error rates were 0.2906, 0.3270, and 0.3233 for SRER, EER_CS, and EER_RS, respectively, with biases of 0.0740, 0.0376, and 0.0413, respectively.

**Table 3:** Median error rates in ENCODE data using shadow linear regression and smoothing spline approaches^a^

Samples	Expected ER	SRER	SRER Bias	EER_CS	EER_CS Bias	EER_RS	EER_RS Bias
SRR002053	0.5548	0.4153	0.1395	0.4609	0.0939	0.4565	0.0983
SRR002056	0.3646	0.2906	0.0740	0.3270	0.0376	0.3233	0.0413
SRR002065	0.4578	0.3371	0.1207	0.3740	0.0838	0.3701	0.0877
SRR005092	0.6300	0.4047	0.2253	0.4797	0.1503	0.4727	0.1573
SRR005093	0.4839	0.3928	0.0911	0.4221	0.0618	0.4173	0.0666

^a^Based on 1000 replicates. The frequency-based simulation approach was applied. For each replicate, we considered the top 1000 reads with the highest frequencies as the error-free reads and generated 1000 pairs of error-free read counts and shadow counts

*Expected ER* expected error rate in simulation studies

*SRER* error rate estimated using shadow regression

*SRER Bias* the absolute value of the difference between SRER and Expected ER

*EER_CS* empirical error rate estimated using cubic smoothing spline

*EER_CS Bias* the absolute value of the difference between EER_CS and Expected ER

*EER_RS* empirical error rate estimated using robust smoothing spline

*EER_RS Bias* the absolute value of the difference between EER_RS and Expected ER

### Simulation results for PhiX DNA data

Table 4 reports the median error rates obtained using different approaches based on the next-generation sequencing data from two PhiX DNA samples. For both samples, the smoothing spline approaches provided more accurate estimations of the error rates than did shadow linear regression. For example, in sample 100217, the median of the expected error rate in the simulation was 0.0323. Using SRER, the median of the estimated error rate was 0.0250, with a bias of 0.0073 compared to the expected error rate. The median estimated error rate for EER_CS and EER_RS was 0.0315, resulting in a much smaller bias of 0.0008 for both approaches.

**Table 4:** Median error rates in PhiX DNA data using shadow linear regression and smoothing spline approaches^a^

Samples	Expected ER	SRER	SRER Bias	EER_CS	EER_CS Bias	EER_RS	EER_RS Bias
100217	0.0323	0.0250	0.0073	0.0315	0.0008	0.0315	0.0008
100514	0.0152	0.0143	0.0009	0.0152	0.0000	0.0152	0.0000

^a^Based on 1000 replicates. The frequency-based simulation approach was applied. For each replicate, we considered the top 1000 reads with the highest frequencies as the error-free reads and generated 1000 pairs of error-free read counts and shadow counts

*Expected ER* expected error rate in simulation studies

*SRER* error rate estimated using shadow regression

*SRER Bias* the absolute value of the difference between SRER and Expected ER

*EER_CS* empirical error rate estimated using cubic smoothing spline

*EER_CS Bias* the absolute value of the difference between EER_CS and Expected ER

*EER_RS* empirical error rate estimated using robust smoothing spline

*EER_RS Bias* the absolute value of the difference between EER_RS and Expected ER

### Next-generation sequencing data analysis results

We applied our smoothing spline approaches to evaluate the error rates for the next-generation sequencing data from MAQC, mutation screening, ENCODE and PhiX DNA samples, and compared error rates using our smoothing spline approaches and shadow linear regression. The estimated error rates are reported in Tables 5, 6, 7 and 8, respectively, for samples from the MAQC, mutation screening, ENCODE, and PhiX DNA data sets. From the results we can observe that the smoothing spline approaches always provided relatively higher estimates of the error rates. For example, in sample SRR037454 from MAQC (Table 5), the estimated error rates were 0.1596, 0.2041, and 0.2196, respectively, for SRER, EER_CS, and EER_RS. This was expected given that shadow linear regression tended to underestimate the error rates in the simulation results.

**Table 5:** Error rates in real MAQC data using shadow linear regression and smoothing spline approaches

Samples	SRER	EER_CS	EER_RS
SRR037452	0.2695	0.3124	0.3362
SRR037453	0.1598	0.1819	0.1822
SRR037454	0.1596	0.2041	0.2196
SRR037455	0.1482	0.1694	0.1700
SRR037456	0.1657	0.2062	0.2162
SRR037457	0.1541	0.1796	0.1793
SRR037458	0.2386	0.2573	0.2575
SRR037459	0.1996	0.2216	0.2233
SRR037460	0.2027	0.2504	0.2647
SRR037461	0.1779	0.2058	0.2093
SRR037462	0.1858	0.2329	0.2319
SRR037463	0.1771	0.2019	0.2072
SRR037464	0.1850	0.2377	0.2448
SRR037465	0.1842	0.2019	0.2070

*SRER* error rate estimated using shadow regression

*EER_CS* empirical error rate estimated using cubic smoothing spline

*EER_RS* empirical error rate estimated using robust smoothing spline

**Table 6:** Error rates in real mutation screening data using shadow linear regression and smoothing spline approaches

Samples	SRER	EER_CS	EER_RS
SRR032565	0.0753	0.1167	0.1206
SRR032566	0.0584	0.0746	0.0745
SRR032567	0.0846	0.1420	0.1446
SRR032568	0.0686	0.1566	0.1566
SRR032569	0.0597	0.1015	0.1020
SRR032570	0.0691	0.0973	0.0954
SRR032571	0.0724	0.1400	0.1400
SRR032572	0.0818	0.1611	0.1593
SRR032573	0.1602	0.2756	0.2752
SRR032574	0.0557	0.1004	0.1053
SRR032575	0.0882	0.1191	0.1179
SRR032576	0.1101	0.1915	0.1899
SRR032577	0.0762	0.1282	0.1280
SRR032578	0.1365	0.1874	0.1873
SRR032580	0.0727	0.1262	0.1266
SRR032581	0.0981	0.0506	0.0511
SRR032582	0.0941	0.1689	0.1679
SRR032583	0.1141	0.2057	0.2042
SRR032584	0.0849	0.0963	0.0742
SRR032586	0.0623	0.3606	0.3621
SRR032587	0.0857	0.1524	0.1532
SRR032588	0.0701	0.1446	0.1440
SRR033543	0.0802	0.1084	0.1102
SRR033544	0.1175	0.1588	0.1586

*SRER* error rate estimated using shadow regression

*EER_CS* empirical error rate estimated using cubic smoothing spline

*EER_RS* empirical error rate estimated using robust smoothing spline

**Table 7:** Error rates in real ENCODE data using shadow linear regression and smoothing spline approaches

Samples	SRER	EER_CS	EER_RS
SRR002053	0.4134	0.4469	0.4453
SRR002056	0.3225	0.3020	0.3355
SRR002065	0.3842	0.3918	0.3913
SRR005092	0.4628	0.4884	0.4776
SRR005093	0.4090	0.4724	0.4013

*SRER* error rate estimated using shadow regression

*EER_CS* empirical error rate estimated using cubic smoothing spline

*EER_RS* empirical error rate estimated using robust smoothing spline

**Table 8:** Error rates in real PhiX DNA data using shadow linear regression and smoothing spline approaches

Samples	SRER	EER_CS	EER_RS
100217	0.0261	0.0317	0.0315
100514	0.0142	0.0155	0.0157

*SRER* error rate estimated using shadow regression

*EER_CS* empirical error rate estimated using cubic smoothing spline

*EER_RS* empirical error rate estimated using robust smoothing spline

## Discussion

Due to its high-throughput capacity and low cost, next-generation sequencing has been widely used to address a diverse range of biological problems. However, because the higher error rates in next-generation short-read sequencing data can impact the downstream genomic analyses, it is critical to accurately assess these error rates before genomic analyses are performed. We have proposed an empirical approach that more accurately estimates error rates for next-generation, short-read sequencing data than other available approaches.

In this paper, we first reviewed the shadow linear regression approach for short sequencing read error rate estimation proposed by Wang et al. [7]. The shadow regression approach was developed based on the assumption of a linear relationship between the number of reads sequenced and the number of shadows. The linear assumption may be appropriate if one could plot the counts of true error-free reads and associated shadow counts obtained using only the reads containing errors. However, the sequencing data is noisy and such information is not identifiable. Therefore, the proposed approach to compute a sample-level error rate using the median of the error rates obtained at different values of error-free read counts and corresponding fitted shadow counts is likely to be more robust. From the sample next-generation sequencing data presented in the Wang et al. paper [7], the linear assumption for the read-shadow relationship might hold for the PhiX DNA sequencing data, but it is not valid for the mRNA sequencing data (MAQC and ENCODE) or the mutation screening re-sequencing data (Additional file 1). Therefore, we employed smoothing spline approaches to model the nonlinear read-shadow relationship and proposed an empirical approach to estimate the short-read sequencing error rates. The smoothing spline approaches can control the smoothness through the tuning parameter in the penalty term instead of the number and location of knots, which provides the same fit with fewer parameters, and in turn, reduces the likelihood of overfitting the data [25]. Although one could use the linear smoothing spline, in our paper we used the cubic smoothing spline because we desired a smoother interpolating function. Moreover, in addition to the cubic smoothing spline, to improve the robustness of the spline, we also used the robust smoothing spline, employing an iterative re-weighted smoothing spline algorithm with the inverse of the absolute value of the residuals as the weights. We then compared the proposed empirical error rate approach with the shadow linear regression approach using simulation studies. The results from the simulation studies showed that the shadow linear regression underestimated error rates while the proposed empirical approach provided more accurate estimations of the error rates. This was true even for the DNA sequencing data, where the linear read-shadow relationship might be valid.

In practice, the true sample genome sequence might not be the same as the reference genome sequence due to polymorphisms or duplications. Therefore, we performed additional simulations with two scenarios: (1) there is one polymorphism for every 1000 base pairs as was assumed in Wang et al. [7]; and (2) there are two base-pair duplications for every 1000 base pairs. It is important to note that these assumptions are specific for simulating the data and are not required for the shadow-based methods. Specifically, based on the reads of sample SRR037440, uniquely mapped to the reference genome with no mismatches, we added one polymorphism or two base-pair duplications per 1000 base pairs and considered the resulting reads as error-free reads. We then added substitution errors based on pre-specified base-specific error rates. In both scenarios, our proposed approach provided accurate results. For example, in the scenario where we assumed that there was one polymorphism per 1000 base pairs, the expected error rate in the simulation was 0.1977. The estimated error rates using the cubic smoothing spline and robust smoothing spline were 0.1973 and 0.1989, respectively. In the scenario where we assumed that there were two base-pair duplications per 1000 base pairs, the expected error rate in the simulation was 0.1728. The estimated error rates were 0.1722 and 0.1761, respectively, using the cubic smoothing spline and robust smoothing spline. These results showed that the proposed approach is not affected by the polymorphisms or duplications as well as the validity of the shadow-based approaches.

We applied the proposed approach to real data from studies of MAQC, mutation screening, ENCODE, and PhiX DNA and compared the results with those obtained using the shadow linear regression approach. The results showed that shadow regression provided relatively low error rates compared to the proposed approach. For example, for the MAQC mRNA sequencing data, our approach yielded error rates between ~17 and ~32 %, whereas shadow regression provided error rates of ~15 to ~27 %. The lower error rates estimated by the shadow regression method could be attributed to the linear assumption, which is not valid in some real data sets, as can be seen by even simple visual inspection (Additional file 1). When analyzing the DNA sequencing data, for which the linear relationship might be valid, the proposed approach provided estimations of error rates similar to those obtained using shadow linear regression.

To better investigate the performance of the proposed approach, we developed a frequency-based simulation approach to capture the nonlinear relationship between the number of reads sequenced and the number of reads that contained errors based on the sequencing reads from real data sets. We compared these two simulation approaches and showed that in the data generated using the Wang et al. simulation approach the shadow counts increased linearly as the error-free read counts increased, whereas in the data generated using our frequency-based simulation approach, the patterns were similar to those in the original sample data (Additional files 3 and 4).

For the proposed empirical error rates, we adapted the original definition of per-read error rate used by Wang et al. [7], which was the proportion of reads containing sequencing errors among all the reads in a sample. Instead of using the slope from the linear model, we defined the empirical error rate as a function of the error-free read count. That is, in a given sample, the error rate could vary on the basis of the number of reads sequenced, which might be practically more robust than the shadow regression in which one fixed error rate is provided for a given sample. We also defined a sample-level error rate using the median of the error rates obtained using different numbers of error-free read counts and corresponding fitted shadow counts.

Both the proposed empirical approach and the shadow regression can be affected by outliers. Therefore, we suggest pre-processing the sequence data before data analysis using standard statistical approaches, such as boxplot rule (i.e., based on the upper and lower quartiles of the data sample distribution), chi-squared test [43], Dixon test [44], and Grubbs’ test [45]. One can also consider alternative approaches that might be more robust to outliers such as the quantile regression [46] or Akima spline [47, 48]. In this study, we showed the application of the proposed approaches to data from several sequencing experiments, such as DNA sequencing, mRNA sequencing and re-sequencing. We also showed that the shadow-based approaches are valid when polymorphisms and duplications are present. Because the shadow-based approaches (i.e., shadow regression and our proposed approaches) are independent of the reference genome, they can be applied to other types of sequencing experiments, such as extensive polymorphisms, isoforms, or the microbiome. In particular, as the approaches consider only the reads that differ from the error-free reads by up to two bases as the shadows (i.e., reads with errors), the reads with many differences, such as extensive polymorphisms, isoforms or microbiome data, will not be counted as shadows in the analysis.

## Conclusion

In summary, we proposed an empirical error rate estimation approach in which cubic and robust smoothing splines were used to model the read-shadow relationship. The proposed approach does not assume a linear relationship between the error-free reads and shadows counts and provides more accurate estimations of error rates for next-generation, short-read sequencing data.

### Availability of data and materials

The datasets of MAQC, re-sequencing and ENCODE used for the analyses described in this paper were obtained from National Center for Biotechnology Information Sequence Read Archive (Accession numbers: SRX016366 and SRX016368 for MAQC; SRX000570 for ENCODE; and SRX012886, SRX012887, SRX012888, SRX012889, SRX012890, SRX012891, SRX012892, SRX012893, SRX012894, SRX012895, SRX012896, SRX012897, SRX012898, SRX012899, SRX012900, SRX012901, SRX012902, SRX012903, SRX012904, SRX012905, SRX012906, SRX012907, SRX012908, and SRX012909 for mutation screening). The bacteriophage PhiX 174RF1 (PhiX) DNA data were generated by the Center for Cancer Computational Biology at Dana-Farber Cancer Institute and provided by Drs. Xin Victoria Wang and Giovanni Parmigiani, Dana-Farber Cancer Institute. The data supporting the results of this article are included within the article and its additional files: Additional files 1, 2, 3 and 4 which are referenced in the main text.

## Additional files

Additional file 1: Sample sequencing data from MAQC, mutation screening re-sequencing, ENCODE, and PhiX DNA data sets. (DOCX 562 kb) Additional file 2: Read counts for the SRR032577 sample in the mutation screening re-sequencing study. (DOCX 17 kb) Additional file 3: Sample SRR037440 from the MAQC brain experiment 2 data set and corresponding simulated data using frequency-based and Wang et al. simulation approaches. (DOCX 879 kb) Additional file 4: Comparisons of different simulation approaches. (DOCX 20 kb)
