# Supplementary material for: Empirical estimation of sequencing error rates using smoothing splines
Source: BMC Bioinformatics. 2016 Apr 22;17:177. doi: 10.1186/s12859-016-1052-3 (PMC4840868; doi:10.1186/s12859-016-1052-3)

**Figure S1. Sample sequencing data from MAQC, Mutation Screening Re-Sequencing, ENCODE, and PhiX DNA data sets**


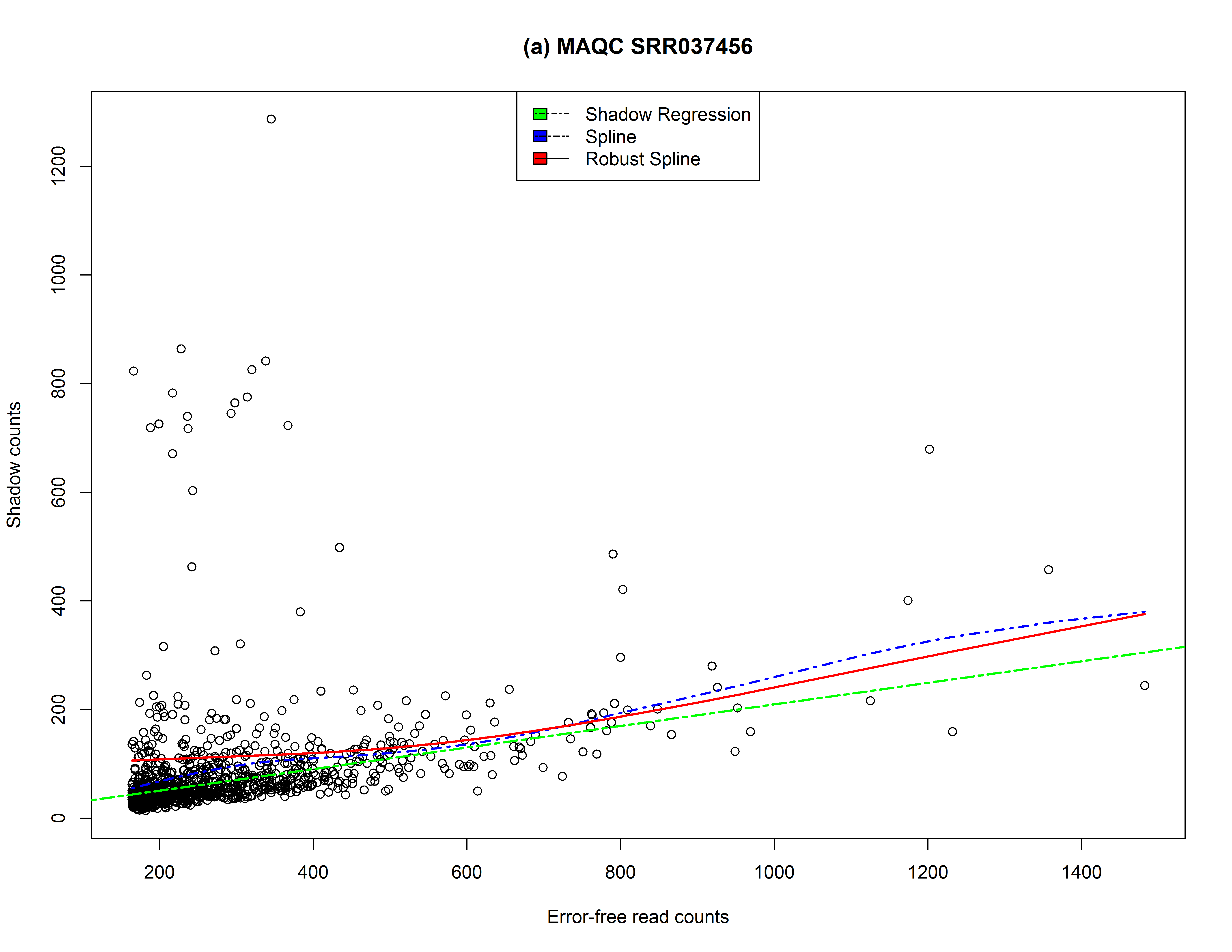

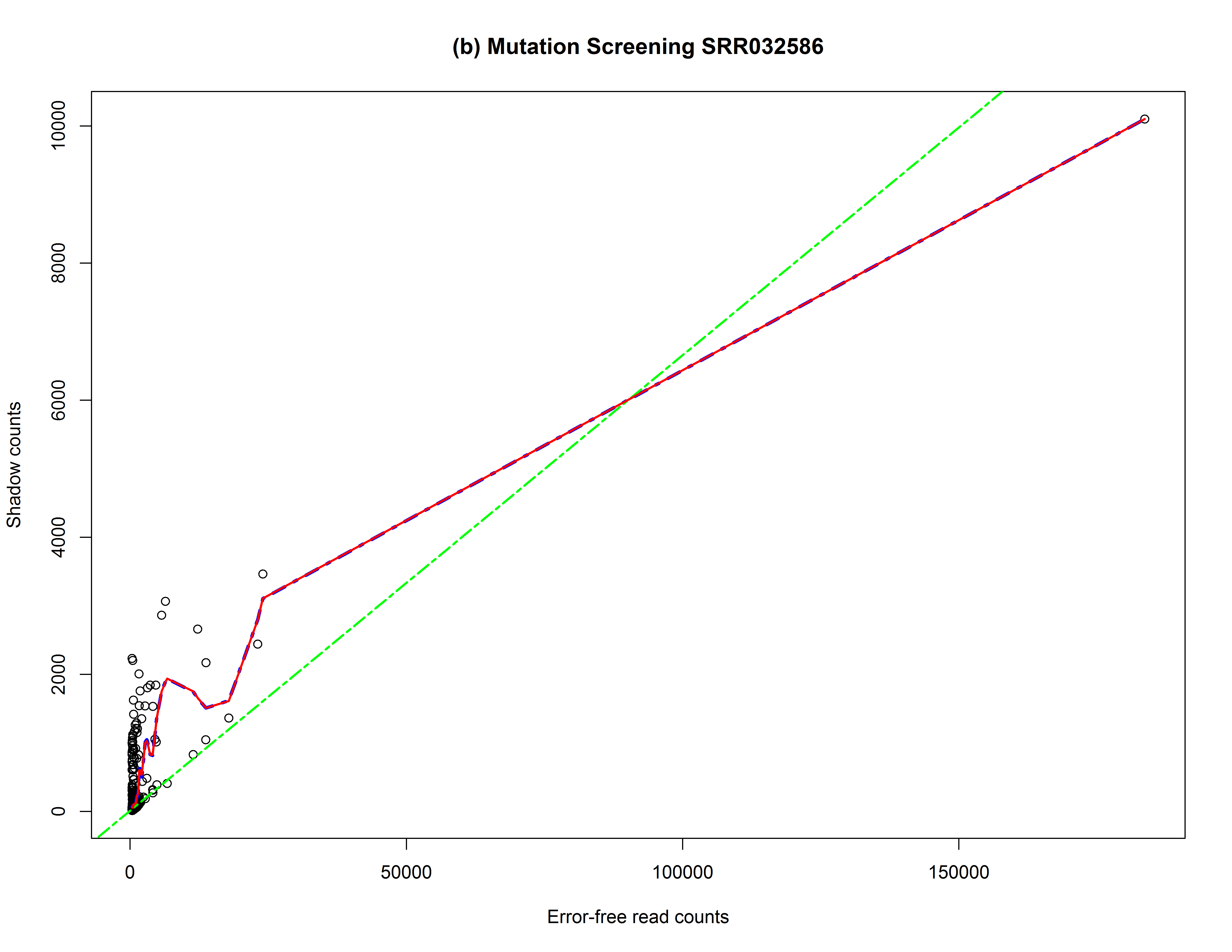


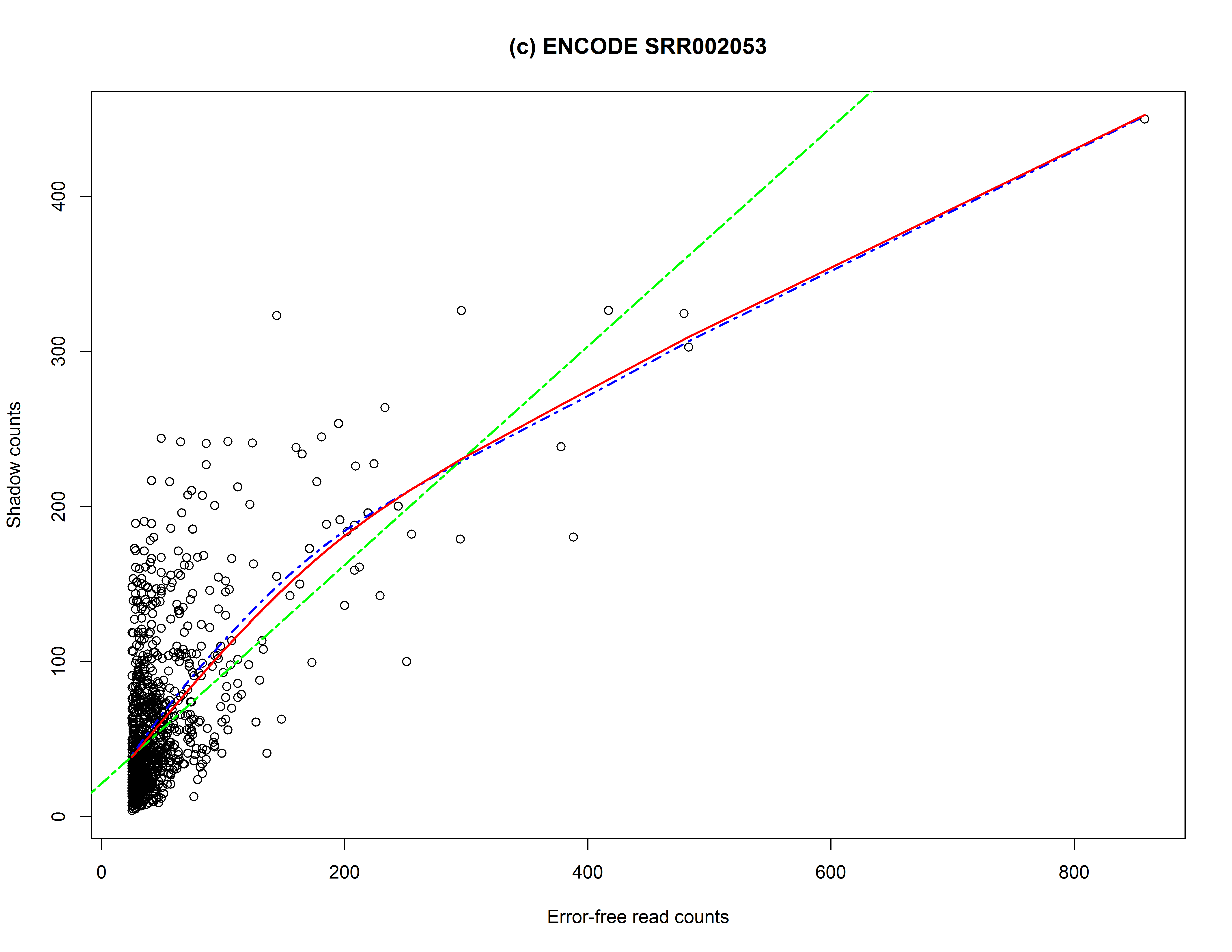

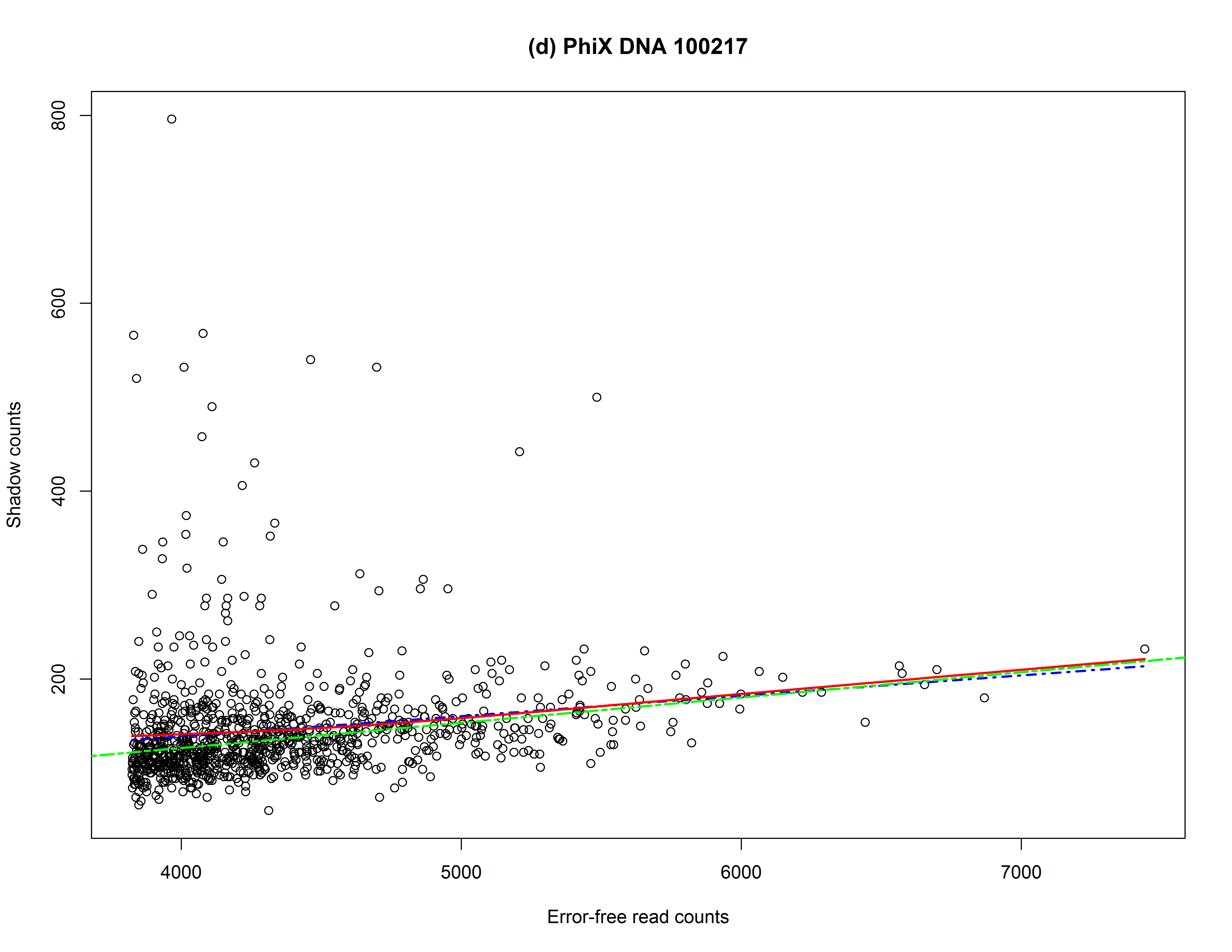

Supplement: Additional file 1: — Sample sequencing data from MAQC, mutation screening re-sequencing, ENCODE, and PhiX DNA data sets. (DOCX 562 kb) [file 12859_2016_1052_MOESM1_ESM.docx]
