# Supplementary material for: Empirical estimation of sequencing error rates using smoothing splines
Source: BMC Bioinformatics. 2016 Apr 22;17:177. doi: 10.1186/s12859-016-1052-3 (PMC4840868; doi:10.1186/s12859-016-1052-3)
Supplement: Additional file 2: — Read counts for the SRR032577 sample in the mutation screening re-sequencing study. (DOCX 17 kb) [file 12859_2016_1052_MOESM2_ESM.docx]

**Figure S2. Read counts for the SRR032577 sample in the mutation screening re-sequencing study**

CAAAGCAACTTATGGGACTTGGTTGGCTTCTGTTTG 22010

CCAGACTTCTCCTCAAGTTATGCAAATCTTATGTCA 17456

GATCGGAAGAGCTCGTATGCCGTCTTCTGCTTGAAA 14515

ACTCATACAGGAGAGAAACCTTATGAATGCAGAGAC 13720

GAGACAGAGCTGTAGAGAAACAAAAAGAGAAAGATG 12759

GATCGGAAGAGCTCGTATGCCGTCTTCTGCTTAGAT 9832

GAACAAATGCTTTTCCAACCCATGAGTGCTAAGAGC 9192

CTCGCCATGATTATTTGACAAATAATGAGACTAGTA 7583

GTATTCATCAAGCTGACTGGATCCATTTGTCCGGGT 7357

GATGACAATCTCCAAGTGACATTTCACTGTGTTCCT 7039

AGATCGGAAGAGCTCGTATGCCGTCTTCTGCTTGAA 6887

GGTATCTAATAGCAGGGAGGAGGAAGGCCTGTTGCT 6846

AGCAACATGCTATGAACAAAGACCCTTCTGTAACAA 6700

GGTGATGTCCTATCACACTAAACATCGATTGGAGTG 6399

AAGAAGCAGTTTTAGCTTCTTAGATCGGAAGAGCTC 6224

GATAATTGAGCTGTGCATGAATGTTAACTCTTAAAA 6136

GGTAGTGGATCTTTCTGTCTATCAAGAACAGGCCTG 6022

GGTATGAGCTTGACTGAGAGGCCTCTCCTGACTATC 5894

ATCACAGAATCACAAAGCTGGTAAGGAGCCTCCCAA 5702

AGCAAACTGAAATTTTCCCATACTGAGTCATTCATG 5670

GCAGTTCTAGTCAAGCATACAATATCCAGAACCACC 5599

GTACATAGTAGGTGCTCGATGAATGGTTATTTCCTC 5596

AATTTTATGACCAGGCAGTGTTAAAATTAATACTTA 5489

GTGCTCTTTCTTGAGAAGTCTTCCAGTTATTCAACT 5361

GTTACACAACACATGTCAGTTGCAGGGGCAGGAACT 5279

GATTCTCTTTGCCAGCTTAGATGGCTTCGGTTTCAG 5193

GGTATATGATGGCTCAGGATGCTTTTTCCTCCTTTT 5190

ATCTGTGACTAGGACACTTCCACCCACCCAGTCGAT 5187

GATAGGCAGATGTTGTCTTGCAAAACGAGCAAACAG 4947

GTACTTCAATGTCCCTCTCTATAACTTGGACCATGA 4900

GATAGATTGTTCCAGCCTTGCCTGGCTCCCCTGTGA 4868

ATCACCATGATTCCAAACACACTGACGGGGATGCAG 4837

GAATGCATGAGTGAATCCATTTGTGAGAGAGCGAAT 4829

AGCAAAGATCAAACTCCAAGAAATGCTCCTCTGTGA 4738

GGACATGGTAGAATGCCTGCGGAACAAGAACTACAA 4732

GGTAATGAATTAAGGCTGGGAGCAACTTGACTTTGG 4710

GGGTGTTCAGAAACTTACAGATTCGTGGTGAAGGAC 4605

GGAATCTAGGAAACAGGACTGGACTTGGTATCTGAG 4598

GTTAACTCAAGAGCACAACAGAAGTACCAGAGCACC 4578

AACTGCTCTATTCCTTAGCAACCTTTGTTCTTTTTA 4463

ATAGTTCTAAGAGCTGACCCTTGCCTATGACAGCAT 4452

……
