# Supplementary material for: Empirical estimation of sequencing error rates using smoothing splines
Source: BMC Bioinformatics. 2016 Apr 22;17:177. doi: 10.1186/s12859-016-1052-3 (PMC4840868; doi:10.1186/s12859-016-1052-3)

**Figure S3. Sample SRR037440 from the MAQC brain experiment 2 data set and corresponding simulated data using frequency-based and Wang et al. simulation approaches**


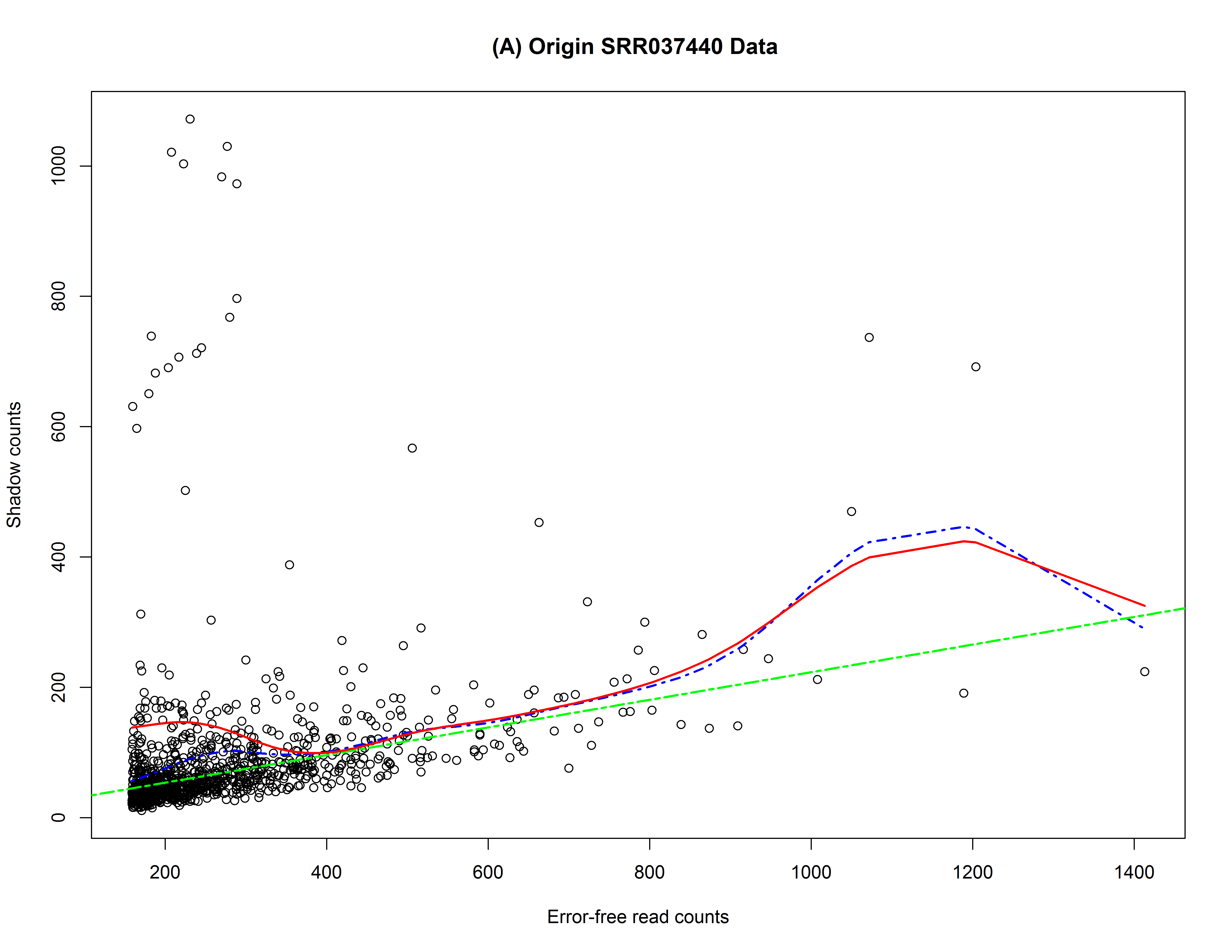

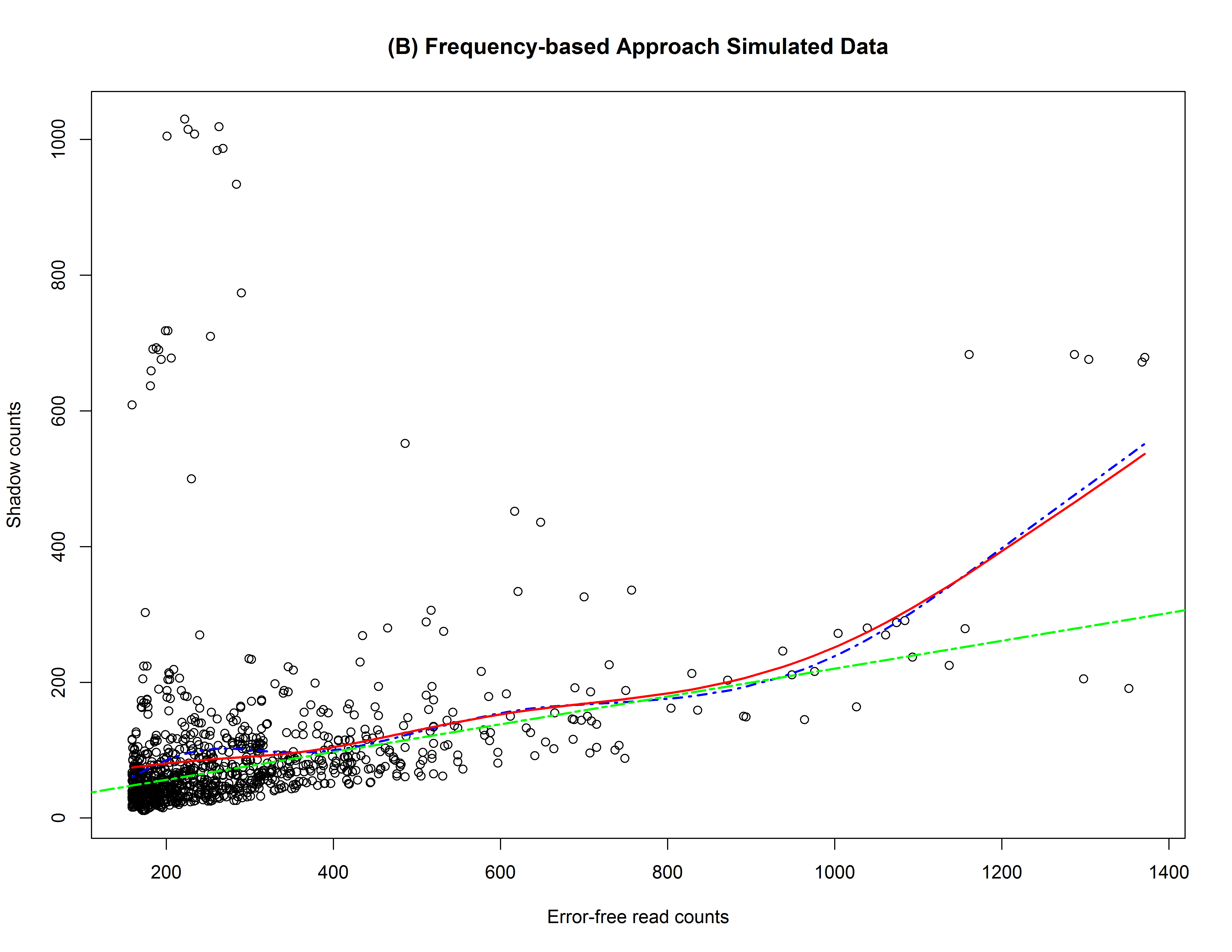


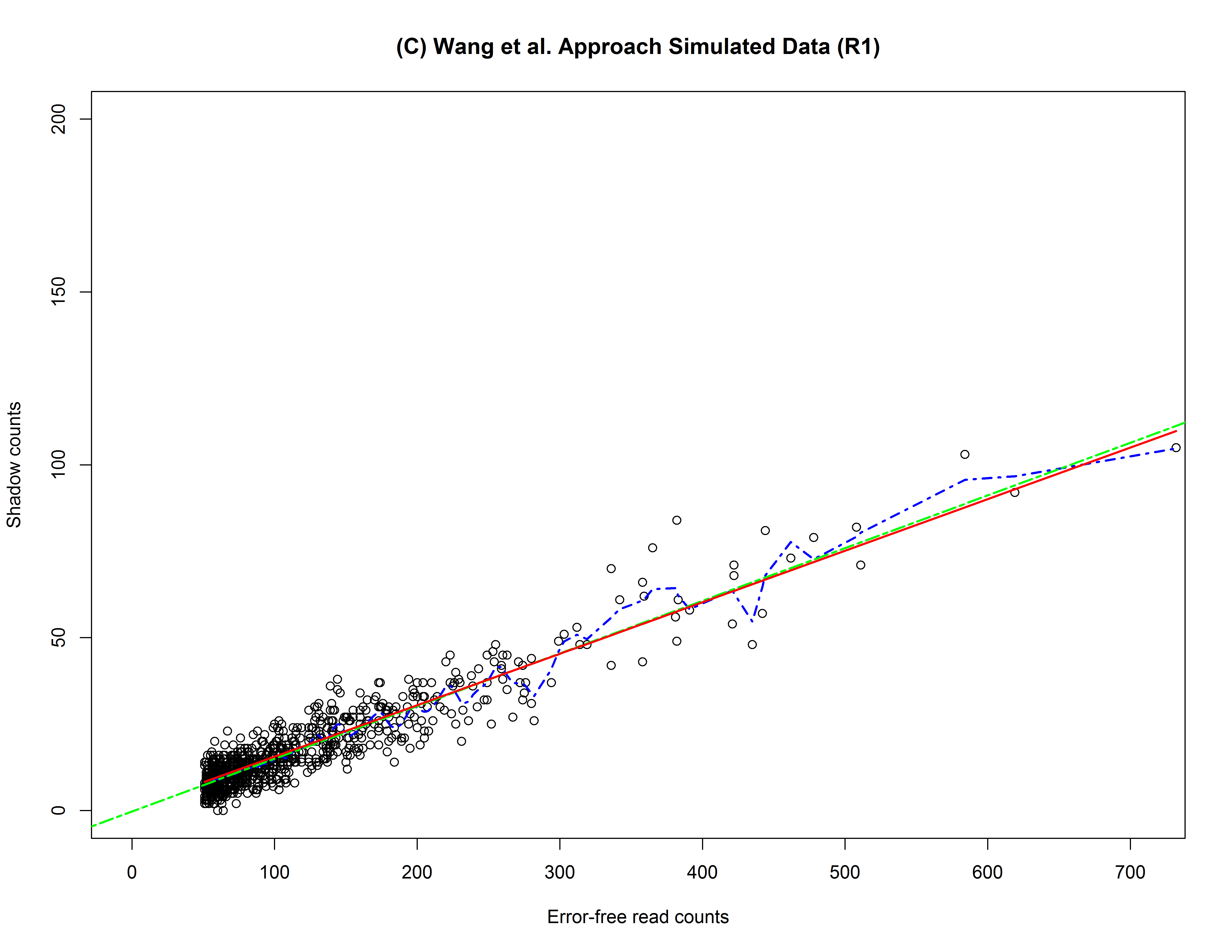

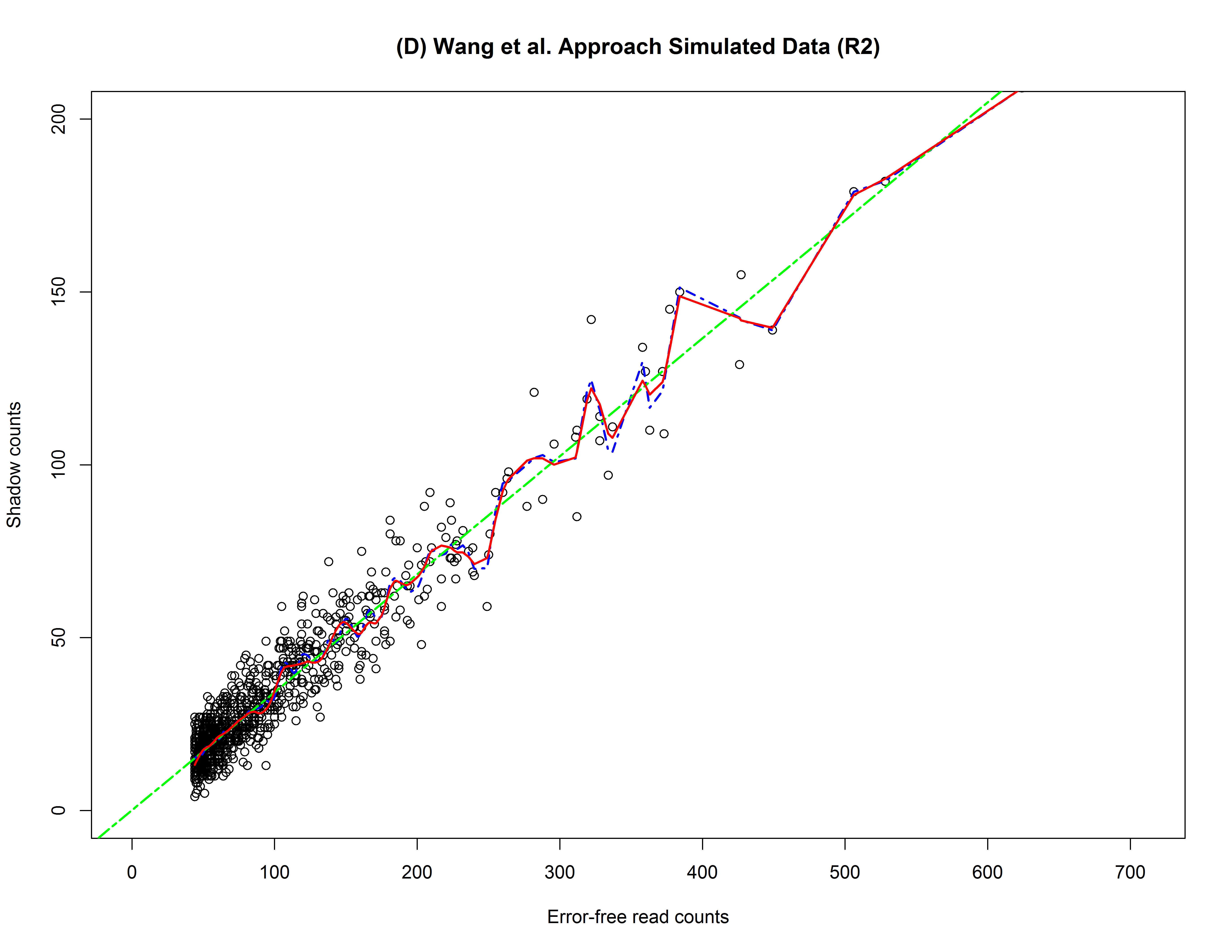


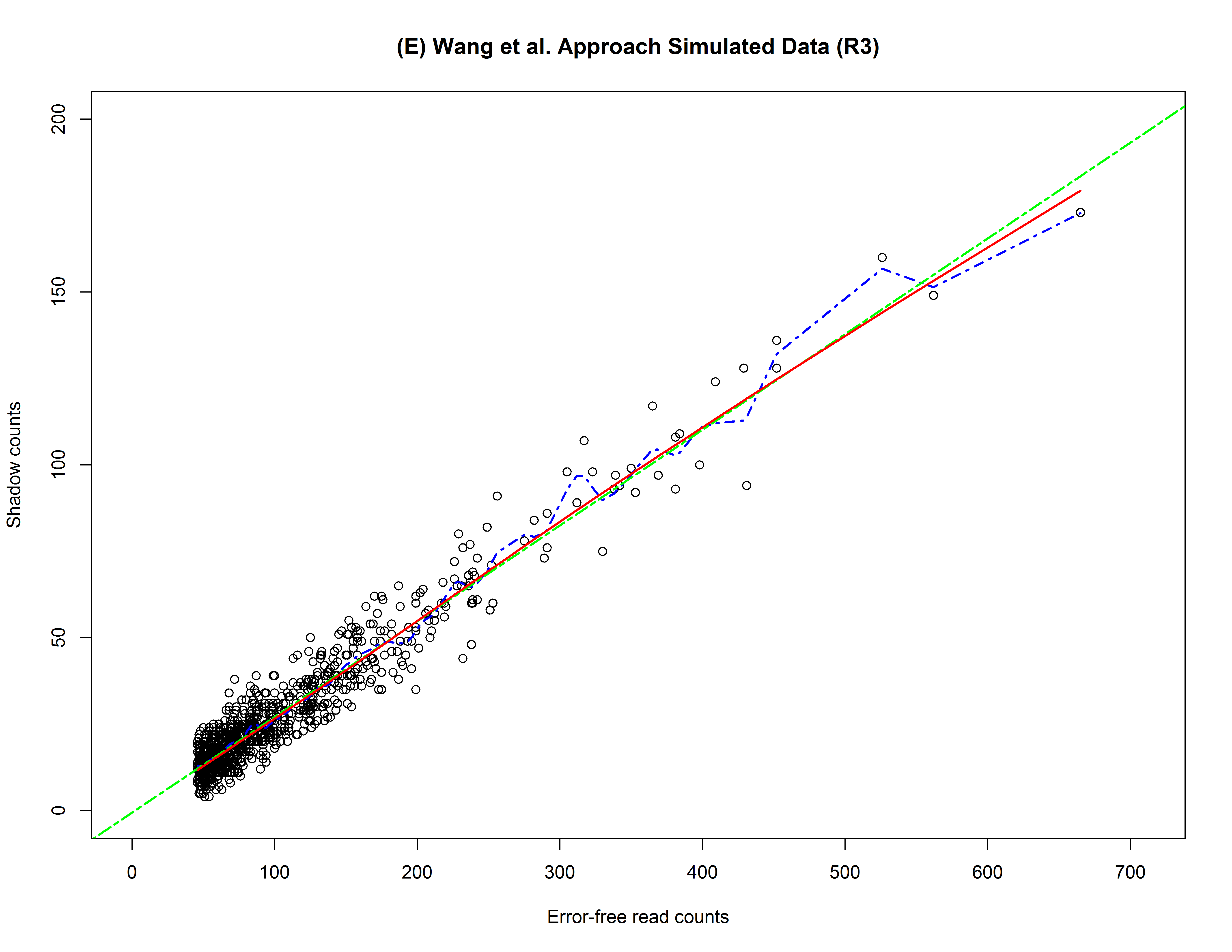

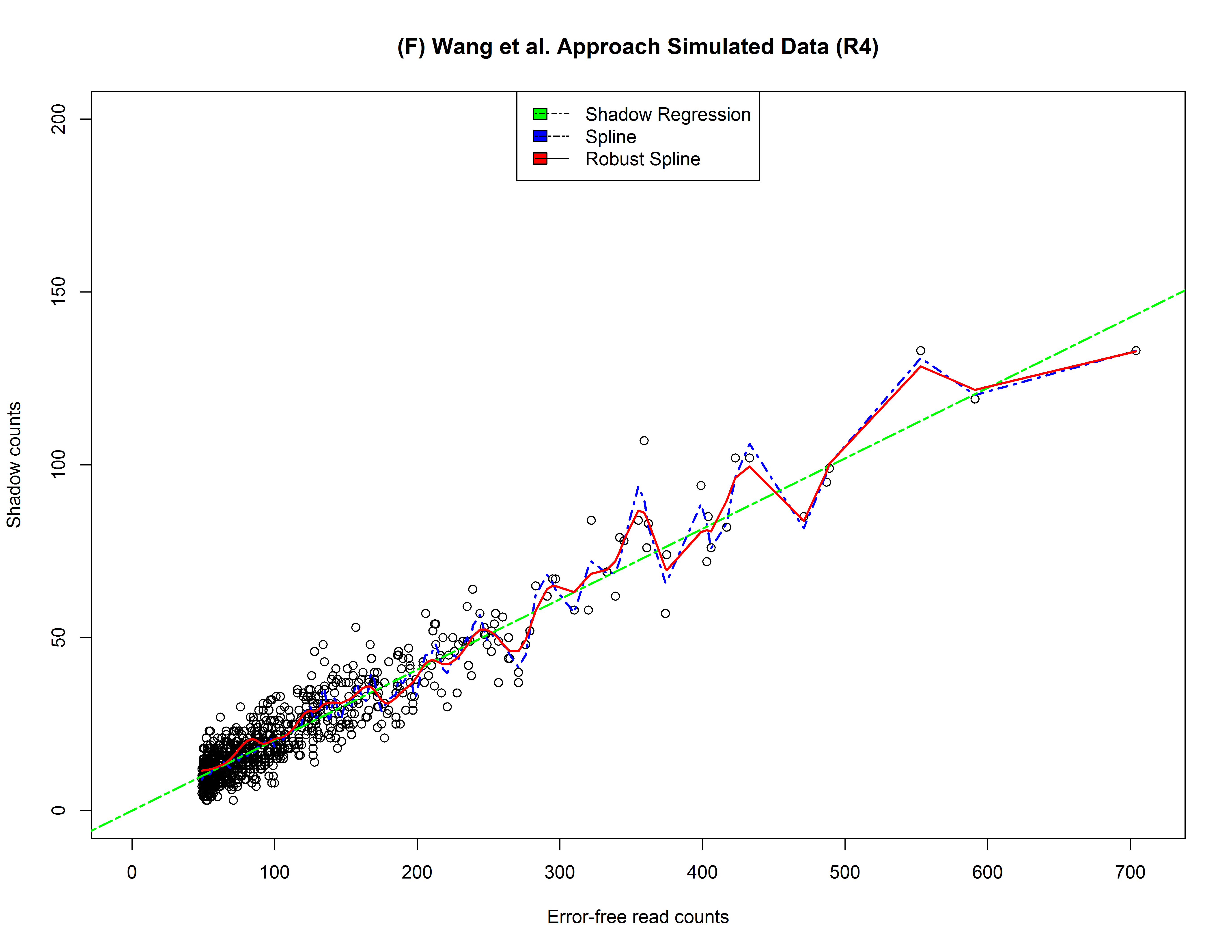

Supplement: Additional file 3: — Sample SRR037440 from the MAQC brain experiment 2 data set and corresponding simulated data using frequency-based and Wang et al. simulation approaches. (DOCX 879 kb) [file 12859_2016_1052_MOESM3_ESM.docx]
