# Supplementary material for: Empirical estimation of sequencing error rates using smoothing splines
Source: BMC Bioinformatics. 2016 Apr 22;17:177. doi: 10.1186/s12859-016-1052-3 (PMC4840868; doi:10.1186/s12859-016-1052-3)
Supplement: Additional file 4: — Comparisons of different simulation approaches. (DOCX 20 kb) [file 12859_2016_1052_MOESM4_ESM.docx]

***Comparisons of Different Simulation Approaches***

We performed a simulation study to compare the Wang et al. simulation approach with our proposed frequency-based simulation approach using information from sample SRR037440 in Wang et al.’s study [7]. Figure S3 shows the relationships between the shadow counts and error-free read counts for the original SRR037440 sample data (panel A), as well as the simulated data generated using the frequency-based simulation approach (panel B) and the Wang et al. simulation approach using different pre-specified base-specific error rates ( as described in the Method; panels C-F). As expected, the data generated using the Wang et al. simulation approach showed that the shadow counts increase linearly as the read counts increase, which does not reflect the true pattern of the original data (panel A). Also, different pre-specified base-specific error rates had very similar performance. On the other hand, the data generated using the frequency-based simulation approach (panel B) showed a pattern very similar to that of the original data. We also plotted the fitted shadow linear regression lines, cubic smoothing spline curves, and robust smoothing spline curves in Figure S3 for the original and simulated data, respectively. It should be noted that the shadow linear regression lines and the smoothing spline curves performed similarly when the data were generated using the Wang et al. simulation approach because the approach generated data using a linear relationship. However, compared to the linear lines, the smoothing spline curves captured more features when both the original data and the data generated using the frequency-based simulation approach were used (panels A and B).

We have also evaluated the error rates for the original sample SRR037440 (Table S1) and the simulated data generated using different simulation approaches and different error rate estimation approaches, including shadow linear regression (SRER), cubic smoothing spline (EER_CS), and robust smoothing spline (EER_RS) approaches. When the data sets were generated using the Wang et al. approach, the estimated error rates obtained using the different approaches were very similar and were close to the expected error rate, which was consistent with the results shown in Figure S3. For example, when the first pre-specified base-specific error rate (R1) was used, the expected per-read error rate was 0.1308 and the estimated error rates were 0.1313, 0.1328, and 0.1319 for SRER, EER_CS, and EER_RS, respectively. In the data set generated using the frequency-based simulation approach, the approaches using smoothing splines provided more accurate estimations for the per-read error rates than the shadow linear regression approach: the expected error rate was 0.2436, and the estimated error rates were 0.1614, 0.2157, and 0.2166 for SRER, EER_CS, and EER_RS, respectively.

**Table S1. Comparison of the Wang et al. and proposed frequency-based simulation approaches using sample SRR037440***

| **Simulation Approaches** | | **Expected ER** | **SRER** | **EER_CS** | **EER_RS** |
| --- | --- | --- | --- | --- | --- |
|  |  |  |  |  |  |
| **Original sample** | | - | 0.1910 | 0.2297 | 0.2409 |
|  |  |  |  |  |  |
| **Wang et al.** | **R1** | 0.1308 | 0.1313 | 0.1328 | 0.1319 |
|  | **R2** | 0.2563 | 0.2548 | 0.2560 | 0.2553 |
|  | **R3** | 0.2135 | 0.2149 | 0.2173 | 0.2153 |
|  | **R4** | 0.1702 | 0.1692 | 0.1704 | 0.1718 |
| **Frequency_based** | | 0.2436 | 0.1614 | 0.2157 | 0.2166 |
|  |  |  |  |  |  |

* **For the frequency-based simulation approach, we considered the top 1,000 reads of sample SRR037440 with the highest frequencies as the error-free reads and generated 1,000 pairs of error-free read and shadow counts.**

Expected ER: expected error rate in simulation studies

SRER: error rate estimated using shadow regression

EER_CS: empirical error rate estimated using cubic smoothing spline

EER_RS: empirical error rate estimated using robust smoothing spline

R1, R2, R3 and R4: different pre-specified base-specific error rates used in the simulation as described in the Methods section
